# Association of Healthcare Professionals’ Characteristics and ZIP-Code-Level Income of Their Practice Location with Routine Assessment of HPV Vaccination Status

**DOI:** 10.3390/vaccines14090801

**Published:** 2026-09-11

**Authors:** Olajumoke Ope Oladoyin, Paula M. Cuccaro, Lara S. Savas, Robert Yu, Sheryl McCurdy, Monalisa Chandra, Joel Fokom Domgue, Sanjay Shete

**Affiliations:** 1 Department of Health Promotion and Behavioral Sciences, UTHealth School of Public Health, Houston, TX 77054, USA; 2 Department of Biostatistics, The University of Texas MD Anderson Cancer Center, Houston, TX 77030, USA; 3 Department of Epidemiology, The University of Texas MD Anderson Cancer Center, Houston, TX 77030, USA; 4 Division of Cancer Prevention and Population Sciences, The University of Texas MD Anderson Cancer Center, Houston, TX 77030, USA

**Keywords:** HPV vaccine, human papillomavirus, HPV vaccine assessment, healthcare professionals, ZIP-code-level income, cancer prevention

## Abstract

Background: Although the human papillomavirus (HPV) vaccine has the potential to prevent most HPV-related cancers, its uptake in Texas remains lower than the national uptake and the Healthy People 2030’s goal of 80% completion among adolescents. The Centers for Disease Control and Prevention (CDC) advises healthcare professionals (HCPs) to assess immunization status at every clinical encounter and to routinely integrate these standards into their practice. Socioeconomic characteristics of individuals influence HPV vaccination; however, the role of HCPs’ individual characteristics and the ZIP-code-level income of their practice location in the routine assessment of HPV vaccination is unknown. Methods: Using data from a survey of HCPs conducted by MD Anderson in Texas in the year 2021, we examined the association between HCPs’ individual characteristics and the ZIP-code-level income of their practice location in HCPs’ routine assessment of HPV vaccination. Results: This study included 1283 HCPs working in Texas. Overall, 37.6% of HCPs reported routinely assessing HPV vaccination status. Compared with HCPs whose practice was based in lower-income communities (median household income below $56,500), those who practiced in higher-income communities had 32% lower odds of routinely assessing HPV vaccination status (aOR: 0.68; 95% CI: 0.48–0.96). Compared to HCPs without prior formal training on HPV vaccination, the odds of HCPs assessing HPV vaccination status routinely were 393% higher (aOR: 4.93; 95% CI: 3.21–7.58) among HCPs who had formally trained less than two years ago, 123% higher (aOR: 2.23; 95% CI: 1.52–3.28) among HCPs formally trained between two and five years ago, and 92% (aOR: 1.92; 95% CI: 1.28–2.87) higher among HCPs formally trained more than five years ago. Conclusions: Our findings underscore the need for targeted interventions and education to promote routine HPV vaccination communication and status assessment among HCPs in higher-income communities in Texas. These efforts should include approaches to address vaccine hesitancy and introduce reminders and system-level prompts. Future research should investigate the contextual and organizational factors that contribute to these income-related differences to better inform implementation strategies.

## 1. Introduction

With a lifetime probability of human papillomavirus (HPV) infection around 80%, HPV remains one of the most common sexually transmitted infections [1]. In the United States (US), HPV infection causes 91% of anal and cervical, 70% of oropharyngeal, 63% of penile, 75% of vaginal, and about 69% of vulvar cancers [2,3]. Among males, the national incidence rate of oropharyngeal cancer increased from 8.9 per 100,000 [4], from 2017 to 18.4 per 100,000 in 2021 [5], and among women from 1.7 per 100,000 in 2017 [4], to 6.7 per 100,000 in 2021 [5]. In 2022, the age-adjusted rate of all HPV-associated cancers in Texas was 12.0 per 100,000 population, and the state ranked fourth nationally in cervical cancer incidence [6].

In 2024, 78.2% of U.S. adolescents ages 13–17 years had initiated HPV vaccination. In contrast, Texas reported a lower initiation rate of 69.9% and a completion rate of 52.2%, ranking 46th nationwide [7,8]. The HPV vaccine can prevent about 90% of HPV-related cancers [2]. This vaccine was approved in 2007, initially recommended for females aged 9–26 years [9]. In 2011, the Centers for Disease Control and Prevention (CDC) revised its recommendations for HPV vaccination, recommending that vaccination begin as early as age nine for both males and females [9]. A two-dose schedule is recommended for individuals who begin vaccination before age 15, with three doses for those who start at or after age 15, and for immunocompromised individuals [10]. The national target for HPV vaccination is to have 80% of adolescents complete the recommended vaccine doses by 2030 [11].

In addition to the overall low uptake in Texas, the rate of HPV vaccine uptake varies across locations within the state [12,13,14], highlighting geographical variation in HPV vaccination [15]. HPV vaccination intent and uptake are influenced by both socioeconomic and contextual factors [12,16,17,18]. At the individual level, factors such as income influence HPV vaccine uptake, with lower odds of completion among adolescents living at or above the federal poverty level [15]. Among higher-income populations, vaccination intention is often associated with concerns about vaccine safety, whereas among lower-income populations, it is more commonly linked to limited knowledge about the vaccine or not receiving a Healthcare Professional’s (HCP’s) recommendation [16]. Additionally, a study found that White parents are more likely to express concern about the number of shots administered during a visit rather than the diseases being prevented, compared with non-White parents [19]. One of the most commonly identified factors influencing HPV vaccine uptake is HCPs’ recommendations [17,20,21,22,23,24,25,26,27]. The CDC recommends that HCPs routinely assess patients’ immunization status and provide strong vaccine recommendations at every clinical encounter [28], underscoring the importance of integrating these practices into standard clinical care. However, assessment practices vary across HCPs. HCPs’ assessments of their patients’ HPV vaccine status are associated with their level of knowledge, prior vaccination training, practice type, and vaccine policy within their practice [29,30].

In Texas, a majority of HCPs reported that they do not routinely assess HPV vaccination status, with lower assessment rates in urban than in rural settings [29], in addition to the wide geographic variation in HPV vaccination across the state [12,13,14] and the differences in HPV vaccination coverage by socioeconomic status [31]. Prior studies have examined the association between provider recommendations and HPV vaccination outcomes [17,20,21,22,23,24,25,26,27]. Due to the increased HPV vaccine hesitancy among parents with higher socioeconomic status [16], HCPs’ vaccination communication and assessment practices may be suboptimal in this population. Also, adolescents from socioeconomically advantaged families have a lower risk of HPV infection [32] and higher-income communities have lower HPV-associated diseases [33]; therefore, HCPs practicing in high-income communities may delay HPV vaccination assessment based on assumptions about the perceived risk of HPV exposure in this population. However, to our knowledge, no study to date has examined whether HCPs’ routine assessments of HPV vaccination status vary by HCPs’ individual characteristics and the ZIP-code-level income of their practice location. Therefore, our hypothesis was that HCPs’ routine assessment of patients’ HPV vaccination status would be associated with the ZIP-code-level median household income of their practice location. Such knowledge could highlight areas requiring targeted interventions and tailored training for HCPs to increase HPV vaccine equity and vaccination rates statewide.

## 2. Methods

This cross-sectional study examined data from a state-wide survey conducted by the University of Texas MD Anderson Cancer Center among HCPs, including physicians, nurse practitioners, and physician assistants, who were between 25 and 75 years old, held valid Texas occupational licenses, and had accessible email addresses in the LexisNexis database, Alpharetta, GA, USA. (https://risk.lexisnexis.com/products/provider-data-masterfile, accessed on 8 September 2026) The LexisNexis Master Provider Referential Database is a centralized, integrated source of public, proprietary, and verified provider data. The database was used to identify HCPs practicing in Texas and to extract available e-mail addresses linked to eligible provider records. Its identity-resolution, deduplication, and data-enrichment capabilities supported more accurate and comprehensive provider identification than reliance on a single registry or licensing source [34]. The data were collected in English via an online platform using REDCap (Version 13.8.5; Nashville, TN, USA) [35,36] between January and April 2021. Up to three follow-up emails were sent to non-respondents, and participants received a $10 gift card as compensation. The research was approved by the MD Anderson Cancer Center Ethical Review Board (IRB No. 2019-1257). A total of 1283 respondents completed the survey, yielding a 7% response rate [29].

Dependent variable: The outcome was an assessment of HPV vaccination status by HCPs in Texas, which was determined with, “At every patient encounter, do you assess HPV vaccination status?” Response options include three categories: Never, Sometimes, Often/Always. Of the survey respondents, 391 (30.5%) reported never assessing HPV vaccination status, 410 (32.0%) reported sometimes assessing HPV vaccination status, and 482 (37.6%) reported often/always assessing HPV vaccination status. “Never” and “sometimes” responses were combined as the assessment is expected to be performed routinely. Those who responded “often/always” are hereafter referred to as those who “routinely” assess HPV vaccination status.

Independent variable: The independent variable was the ZIP-code-level income of the HCPs’ practice location. The 2018–2022 American Community Survey 5-year estimate was used to determine the ZIP-code-level median income [37]. The practice ZIP Code reported by each provider in the survey was linked to the corresponding ACS median household income estimate. The household income at the ZIP-code level was categorized into two groups: below the median (<$56,500) and above the median (≥$56,500).

HCPs-level characteristics include age in years (<35, 35–54, and ≥55), sex (male and female), race (non-Hispanic White, non-Hispanic Black, Hispanic, non-Hispanic Asian, and non-Hispanic Other), number of patients seen per week (1–50, 51–100, and >100), training recency in HPV vaccination (no training, received formal training ≤2 years ago, received formal training between 2 and 5 years ago, and received formal training >5 years ago), number of years in active medical practice (≤10 years, 11–20 years, and >20 years), role (family physician, gynecologist, internal medicine, nurse practitioner/advanced nurse practitioner, pediatrician, physician assistant, and other), and facility type (city/county/public healthcare facility, employed physician practices, FQHC/public facility, faith-based hospital/clinic, group practice, solo practice, university/teaching hospital, and other).

### Statistical Analysis

We examined the distribution of HPV vaccination status assessment among HCPs using descriptive analysis. A logistic regression analysis was used to estimate the odds of HPV vaccination status assessment among HCPs. Based on previous literature and the study’s relevance, variables were selected a priori and included in the analysis. We examined the association between HCPs’ individual characteristics and the ZIP-code-level income of their practice locations in HCPs’ routine assessment of HPV vaccination. The logistic regression model was adjusted for HCPs’ age, sex, race/ethnicity, number of patients seen per week, recency of HPV vaccination training, number of years in active medical practice, physician role, and facility type. The analysis was conducted with SAS software (Version 9.4; SAS Institute Inc., Cary, NC, USA) [38], with statistical significance set at a two-sided *p*-value < 0.05.

## 3. Results

### 3.1. Characteristics of the Study Population

The study included 1283 HCPs: 12.7% were below 35 years, 62.4% were ages 35 to 54 years, 24.9% were 55 years and older, 23.5% were male, 52.6% were non-Hispanic White, 12.8% were Hispanic, and 9.1% were non-Hispanic Black. Nearly half (47.9%) saw fewer than 50 patients per week, and 39.6% saw 51–100 patients per week. Over half of HCPs (57.3%) had no prior formal training on HPV vaccination, and 13.2% had received formal training more than five years ago. One-third (32.3%) were nurse practitioners, 21.4% were physician assistants, 18.9% were pediatricians, and 14.9% were family physicians. Workplace included group practices (32.3%), solo practices (11.3%), and FQHC/public facilities (7.0%). Most HCPs’ workplaces (75.3%) were in ZIP codes with household incomes of $56,500 or higher (Table 1).

### 3.2. Assessment of HPV Vaccination Status by HCPs in Their Routine Practice

Among the 1283 HCPs, 37.6% reported routinely assessing HPV vaccination status. The proportion of HCPs who routinely assessed HPV vaccination status was higher among those who were 55 years and above (49.2%, 95% CI:43.6–54.8), Hispanic (51.2%, 95% CI: 43.6–58.9), received formal training on HPV vaccination within the past two years (67.1%, 95% CI: 59.9–74.3), pediatricians (69.4%, 95% CI: 63.6–75.2), work in a FQHC/Public Facility (61.1%, 95% CI: 51.0–71.2), saw 51–100 patients weekly (50.6%, 95% CI: 46.2–55.1), and whose workplace was located in a ZIP code with an annual median-household income below $56,500 (49.1%, 95% CI: 43.1–55.1) (Table 1).

In the multivariable logistic regression (Table 2), after adjusting for the covariates, HCPs whose workplace was located in a ZIP code with a median household income of $56,500 and above had 32% lower odds of assessing HPV vaccination status routinely (aOR: 0.68; 95% CI: 0.48–0.96) compared to HCPs whose workplace was located in a ZIP code with a median household income below $56,500.

HCPs aged 35–54 years had 37% lower odds of routinely assessing HPV vaccination status (aOR: 0.63; 95% CI: 0.41–0.96) compared to HCPs aged 55 years and older. Compared to non-Hispanic White HCPs, those who were Hispanic had 121% higher odds of assessing HPV vaccination status routinely (aOR: 2.21; 95% CI: 1.46–3.33).

Compared to HCPs who saw 50 or fewer patients weekly, those who saw 51–100 patients and above 100 patients had 147% (aOR: 2.47; 95% CI: 1.80–3.40) and 107% (aOR: 2.07; 95% CI: 1.27–3.36) higher odds, respectively, of routinely assessing HPV vaccination status. Compared to HCPs without formal training on HPV vaccination, the odds of HCPs assessing HPV vaccination status routinely were 92% higher (aOR: 1.92; 95% CI: 1.28–2.87) among those HCPs who received formal training more than five years ago, 123% higher (aOR: 2.23; 95% CI: 1.52–3.28) among those HCPs who received formal training between two and five years ago, and 393% higher (aOR: 4.93; 95% CI: 3.21–7.58) among those HCPs who received formal training less than two years ago.

Compared to family physicians, nurse practitioners had 42% lower odds (aOR: 0.58; 95% CI:0.37–0.89), physician assistants had 73% lower odds (aOR: 0.27; 95% CI: 0.17–0.45), and pediatricians had 204% higher odds (aOR: 3.04; 95% CI: 1.93–4.78) of assessing HPV vaccination status routinely. Compared to HCPs in university/teaching hospitals, the odds of assessment of HPV vaccination status were 206% higher (aOR: 3.06; 95% CI: 1.63–5.75) among HCPs in FQHC/public facilities and 133% higher (aOR: 2.33; 95% CI: 1.41–3.86) among HCPs in solo practice. In this study, sex and number of years in practice were not significantly associated with HCPs’ assessments of HPV vaccination status.

Finally, because 202 of 1283 HCPs (15.7%) were missing information on household income in the ZIP codes where their workplaces were located, we also conducted a complete-case sensitivity analysis for ZIP-level income; the odds ratios were qualitatively similar. Specifically, after adjusting for the covariates, HCPs whose workplace was located in a ZIP code with a median household income of $56,500 and above had 31% lower odds of assessing HPV vaccination status routinely (aOR: 0.69; 95% CI: 0.48–0.98) compared to HCPs whose workplace was located in a ZIP code with a median household income below $56,500.

## 4. Discussion

This study examined the prevalence and factors associated with HCPs’ routine assessments of HPV vaccination, HCPs’ individual characteristics, and the ZIP-code-level income of their practice location. We found that HCPs practicing in ZIP codes with higher household incomes were less likely to routinely assess patients’ HPV vaccination status. These findings on routine assessment of HPV vaccination are novel and consistent with those on HPV vaccine uptake in Texas [31,39]. For example, studies found that areas with a higher proportion of residents living below the federal poverty level are hotspots for HPV vaccination (higher HPV vaccination uptake over 5 consecutive years), while areas with higher incomes are associated with cold spots in HPV vaccination (lower vaccination uptake) [31], and adolescents living in households above the poverty level have been shown to have lower HPV vaccination uptake [39]. Our results suggest that area-level economic factors may be associated with HCP vaccination assessment practices. Such reduced assessment in higher-income settings may partially contribute to the documented pattern in which adolescents from low-income households exhibit higher HPV vaccination uptake than their counterparts in high-income households [40] and adolescents in high-poverty areas have higher HPV vaccination uptake than those in low-poverty areas [41,42].

Another factor that can explain this result is the increased funding and availability of public health programs such as the Vaccines for Children (VFC) program in low-income or medically underserved communities [43]. These programs reduce cost and distance barriers and may encourage HCPs to assess vaccination status and recommend vaccination, knowing it is available at no cost to families. In addition, residents of higher-income areas are more likely to have higher levels of educational attainment, and parents of age-eligible children with higher educational attainment demonstrate lower intent to vaccinate against HPV [16] and higher levels of vaccine hesitancy [44], which may help explain why HCPs are less likely to assess vaccination status in these communities. The findings emphasize the importance of tailored communication strategies or HCP training that addresses vaccine hesitancy among higher-income families while consistently reinforcing the cancer-prevention benefits of HPV vaccination across all families to achieve equitable vaccine uptake.

Another plausible explanation for this result involves patterns in preventive care utilization. Adolescents from families with incomes at or below the federal poverty level are less likely to attend preventive care visits or well-child checkups than their higher-income peers [45,46]. Therefore, HCPs may be more inclined to promote and prioritize vaccination for adolescents in lower-income households because they anticipate fewer opportunities for future visits [47]. Additionally, differences in HCPs’ vaccine assessment practices may reflect variations in their perceptions of patients’ demographic characteristics or vaccine-related attitudes [41,48,49]. For example, a study found that non-Hispanic White parents perceived vaccines as less important for preventing certain illnesses and prioritized the number of injections received at each visit over the number of diseases prevented, compared to non-White parents [19].

In our study, HCPs aged 35–54 years had lower odds of routinely assessing HPV vaccination status. In contrast, higher assessment rates among HCPs aged 55 years and older may reflect greater awareness of HPV-related cancer risk among this population, as cancer diagnosis typically occurs around this age [50]. This awareness may serve as a salient reminder of the long-term consequences of HPV infection, prompting more consistent assessment of vaccination among adolescents. Previous research has shown that younger HCPs tend to have lower self-efficacy in counseling adult or vaccine-hesitant patients, and that years of clinical experience are positively associated with confidence in discussing HPV vaccination [51]. Therefore, the limited experience of younger providers may have contributed to less frequent assessment of vaccination status. Moreover, continuing education has been shown to improve HPV vaccination uptake [52,53], suggesting that strengthening HCP training, particularly among early- and mid-career clinicians, could enhance confidence, assessment practices, and vaccine uptake.

In line with the existing literature, our findings indicate that Hispanic HCPs are more likely to routinely assess HPV vaccination status [29]. Given that HCP recommendations are a key determinant of HPV vaccine uptake, the increased vaccination coverage within this population may be partly attributed to more frequent assessment and recommendation practices among Hispanic HCPs, potentially driven by the higher cancer mortality [54] and the incidence of oropharyngeal and cervical cancers among Hispanic populations [55,56]. Additionally, HCPs’ perceptions of more favorable vaccine attitudes among Hispanic patients, supported by evidence of higher vaccination rates in this group [57] may have further encouraged proactive assessment behaviors.

We also found that HCPs seeing more patients per week were more likely to assess HPV vaccination status. This may reflect greater ability and confidence gained through frequent patient interactions, reinforcing assessment as a routine part of care (as recommended by CDC). Prior research has similarly shown that physicians with larger patient loads are more proactive in their vaccine recommendation practices [58]. Moreover, HCPs with greater clinical experience tend to exhibit greater confidence when discussing HPV vaccination, which, in turn, has been linked to improved vaccine acceptance [59]. This finding highlights the role of HCPs’ experience as an important enabler of HPV vaccine assessment.

Consistent with previous literature [29], our study found that HCPs who received formal training on HPV vaccination were more likely to routinely assess HPV vaccination status than those without such training, and that assessment rates increased with more recent training. Although training recency showed a large effect size, the study did not assess the content, quality, or duration of the training. Limited knowledge of HCPs has been identified as a barrier to delivering HPV vaccine education [60], and the need for additional training is a commonly cited reason for inconsistent assessment practices [61]. These findings emphasize the importance of continuous HCP education on current HPV vaccine guidelines [28], effective communication strategies [62,63], and access to credible vaccine information resources. Strengthening HCPs’ training in HPV vaccination counseling and promotion can foster more consistent assessment practices. Overall, our results highlight the value of regular, HPV-focused continuing medical education across all practice settings to sustain vaccine assessment and recommendation, thereby improving HPV vaccination coverage.

Previous literature had found that pediatricians delivered higher-quality HPV vaccine recommendations than family medicine professionals [64], and are more likely to make presumptive recommendations than other healthcare professionals [65]. Our study found that pediatricians were also more likely to routinely assess HPV vaccination status. An unexpected finding of this study was that nurses were less likely to routinely assess HPV vaccination status. Given nurses’ central role in vaccine delivery and their frequent interactions with patients and parents [66], there is an urgent need for targeted training programs that strengthen HPV vaccine assessment and counseling skills among this cadre of HCPs, and non-pediatric HCPs could benefit from additional communication training.

We also found that HCPs working in FQHCs, public facilities, and solo and group practices were more likely to assess HPV vaccination status than those in university or teaching hospitals, consistent with previous research [29]. This pattern may reflect the influence of public health initiatives such as the Vaccines for Children (VFC) program, which expands vaccine access in low-resource settings [43]. Since FQHCs primarily serve low-income populations, the higher likelihood of assessment in these settings may be linked to evidence showing greater HPV vaccination rates in low-income communities [41,42], possibly driven by increased HCP assessment and recommendation efforts. Compared to other facility types, FQHCs consistently offer HPV vaccination [30], which is indicated to be associated with increased HPV vaccination assessment [29]. This underscores the role of practice setting in promoting routine vaccine assessment. Therefore, improving HPV vaccination uptake may require leveraging practice-level factors that support consistent preventive care. Aligning with CDC guidance to routinely assess immunization status and provide strong recommendations at every encounter [28] reinforces the need to integrate these practices into standard clinical workflows to optimize HPV vaccination coverage.

### Strengths and Limitations

The study’s strengths include providing valuable insights for targeted public health interventions by linking ZIP-level economic data with HCP-level practices. Our approach also enabled the simultaneous examination of multiple variables, such as HCPs’ characteristics and ZIP-level income, thereby facilitating a nuanced understanding of the contextual influences on HCPs’ behavior.

Limitations include a low response rate, which raises the possibility of nonresponse bias, as the survey did not capture responses from HCPs practicing across all geographic areas of Texas. Previous studies have also observed low response rates while conducting research among HCPs in Texas [67,68,69]. Importantly, we did not find significant differences between respondents and non-respondents for provider type or sex, the two factors available for non-respondents. Furthermore, because only HCPs in Texas were surveyed, the study’s findings may not generalize to HCPs in other states. In addition, assessment of HPV vaccination status “at every patient encounter” was self-reported and may have been overestimated due to social desirability or recall bias. However, we sought to minimize this bias by assuring participants that their responses would be kept confidential. Finally, cross-sectional designs do not allow causal inference; therefore, longitudinal or intervention studies are necessary to establish causality. One study limitation was that household income levels among persons attended by HCPs were unknown, making it not possible to assess whether HCPs’ vaccination communication and assessment practices were suboptimal in patients with higher household income levels than in those with low household income levels, overall and in different ZIP-code areas.

## 5. Conclusions

Our findings suggest that area-level income may be associated with HCPs’ engagement in HPV vaccination assessment behaviors in the State of Texas, with potential implications for HPV vaccine uptake and, ultimately, cancer prevention outcomes. The observed pattern may reflect differences in patient populations, competing clinical priorities, or varying institutional emphasis on vaccination assessment across income strata. These differences suggest that economic context not only affects patient-level vaccine behaviors but may also be associated with HCPs’ level of engagement in preventive practices. To inform more effective training programs, future research should explore the influence of training content, quality, or duration. Furthermore, studies should examine strategies to change HCPs’ vaccination attitudes and organizational and system-level factors that contribute to HCPs’ vaccination assessment behaviors.

## Figures and Tables

**Table 1:** Descriptive statistics of HCPs assessment of HPV vaccination status.

Variables	At Every Patient Encounter, Do You Assess HPV Vaccination Status?				
	Total *n* (%)	Never/Sometimes *n* (%)	95% CI	Often/Always *n* (%)	95% CI
	1283	801(62.4%)	59.8–65.1	482 (37.6%)	34.9–40.2
* ZIP-code-Level Median Income					
Less than $56,500	267 (24.7)	136 (50.9)	44.9–56.9	131 (49.1)	43.1–55.1
≥$56,500	814 (75.3)	538 (66.1)	62.8–69.4	276 (33.9)	30.6–37.2
* Age of the provider					
<35	156 (12.7)	114 (73.1)	66.1–80.0	42 (26.9)	20.0–33.9
35–54	764 (62.4)	495 (64.8)	61.4–68.2	269 (35.2)	31.8–38.6
≥55	305 (24.9)	155 (50.8)	45.2–56.4	150 (49.2)	43.6–54.8
* Sex of the provider					
Female	966 (76.5)	604 (62.5)	59.5–65.6	362 (37.5)	34.4–40.5
Male	297 (23.5)	184 (62.0)	56.4–67.5	113 (38.0)	32.5–43.6
Race of the provider					
NH White	675 (52.6)	442 (65.5)	61.9–69.1	233 (34.5)	30.9–38.1
NH Black	117 (9.1)	73 (62.4)	53.6–71.2	44 (37.6)	28.8–46.4
Hispanic	164 (12.8)	80 (48.8)	41.1–56.4	84 (51.2)	43.6–58.9
NH Asian	263 (20.5)	162 (61.6)	55.7–67.5	101 (38.4)	32.5–44.3
NH Others	64 (5.0)	44 (68.8)	57.4–80.1	20 (31.3)	19.9–42.6
* Number of patients seen per week					
1–50	588 (47.9)	442 (75.2)	71.7–78.7	146 (24.8)	21.3–28.3
51–100	486 (39.6)	240 (49.4)	44.9–53.8	246 (50.6)	46.2–55.1
>100	153 (12.5)	80 (52.3)	44.4–60.2	73 (47.7)	39.8–55.6
* Training recency on HPV vaccination					
No formal training	730 (57.3)	543 (74.4)	71.2–77.6	187 (25.6)	22.4–28.8
Received formal training > 5 years ago	168 (13.2)	87 (51.8)	44.2–59.4	81 (48.2)	40.6–55.8
Received formal training 2–5 years ago	211 (16.6)	110 (52.1)	45.4–58.9	101 (47.9)	41.1–54.6
Received formal training ≤ 2 years ago	164 (12.9)	54 (32.9)	25.7–40.1	110 (67.1)	59.9–74.3
* Number of years in practice					
≤10 years	492 (38.7)	335 (68.1)	64.0–72.2	157 (31.9)	27.8–36.0
11–20 years	434 (34.2)	283 (65.2)	60.7–69.7	151 (34.8)	30.3–39.3
>20 years	344 (27.1)	174 (50.6)	45.3–55.9	170 (49.4)	44.1–54.7
Role					
Family Physician	191 (14.9)	103 (53.9)	46.8–61.0	88 (46.1)	39.0–53.2
Gynecologist	69 (5.4)	32 (46.4)	34.6–58.2	37 (53.6)	41.8–65.4
Internal Medicine	22 (1.7)	16 (72.7)	54.1–91.4	6 (27.3)	8.6–45.9
Nurse Practitioner/Advanced Nurse Practitioner	414 (32.3)	285 (68.8)	64.4–73.3	129 (31.2)	26.7–35.6
Pediatrician	242 (18.9)	74 (30.6)	24.8–36.4	168 (69.4)	63.6–75.2
Physician Assistant	274 (21.4)	228 (83.2)	78.8–87.6	46 (16.8)	12.4–21.2
Other	71 (5.5)	63 (88.7)	81.4–96.1	8 (11.3)	3.9–18.6
Facility types or workplace					
City/County/Public HealthCare Facility	49 (3.8)	32 (65.3)	52.0–78.7	17 (34.7)	21.3–48.0
Employed Physician Practices	75 (5.8)	47 (62.7)	51.7–73.6	28 (37.3)	26.4–48.3
FQHC/Public Facility	90 (7.0)	35 (38.9)	28.8–49.0	55(61.1)	51.0–71.2
Faith-Based Hospital/Clinic	27 (2.1)	20 (74.1)	57.5–90.6	7 (25.9)	9.4–42.5
Group Practice	414 (32.3)	227 (54.8)	50.0–59.6	187 (45.2)	40.4–50.0
Solo Practice	145 (11.3)	83 (57.2)	49.2–65.3	62 (42.8)	34.7–50.8
University/Teaching Hospital	404 (31.5)	305 (75.5)	71.3–79.7	99 (24.5)	20.3–28.7
Others	79 (6.2)	52 (65.8)	55.3–76.3	27 (34.2)	23.7–44.7

* 58 observations are missing for age, 20 for sex, 56 for the number of patients seen per week, 13 for years of practice, 10 for training recency, and 202 for ZIP-code level-median income.

**Table 2:** Multivariable logistic regression analyses of HCPs’ assessment of HPV vaccination status.

Variables *	At Every Patient Encounter, Do You Assess HPV Vaccination Status?					
	Crude Odds Ratio			Adjusted Odds Ratio		
	Crude OR	95%CI	*p*-Value	aOR	95%CI	*p*-Value
ZIP-code Median Income						
Less than $56,500	ref			ref		
≥$56,500	0.53	0.40–0.71	<0.0001	0.68	0.48–0.96	0.03
Missing	0.61	0.42–0.89	0.01	0.91	0.56–1.48	0.71
Age of the provider						
≥55	ref			ref		
<35	0.38	0.25–0.58	<0.0001	0.57	0.30–1.07	0.08
35–54	0.56	0.43–0.74	<0.0001	0.63	0.41–0.96	0.03
Missing	0.59	0.33–1.05	0.07	0.71	0.31–1.64	0.43
Sex of the provider						
Female	ref			ref		
Male	1.03	0.78–1.34	0.86	0.72	0.50–1.03	0.07
Missing	0.90	0.35–2.28	0.82	1.72	0.51–5.88	0.39
Race of the provider						
NH White	ref			ref		
NH Black	1.14	0.76–1.72	0.52	1.15	0.68–1.95	0.61
Hispanic	1.99	1.41–2.82	0.0001	2.21	1.46–3.33	0.0002
NH Asian	1.18	0.88–1.59	0.27	1.31	0.90–1.92	0.16
NH Others	0.86	0.50–1.50	0.60	0.75	0.38–1.45	0.39
Number of patients seen per week						
1–50	ref			ref		
51–100	3.10	2.40–4.02	<0.0001	2.47	1.80–3.40	<0.0001
>100	2.76	1.91–4.00	<0.0001	2.07	1.27–3.36	0.003
Missing	1.32	0.72–2.41	0.37	1.65	0.75–3.61	0.21
Training recency on HPV vaccination						
No formal training	ref			ref		
Received formal training > 5 years ago	2.70	1.91–3.82	<0.0001	1.92	1.28–2.87	0.0015
Received formal training 2–5 years ago	2.67	1.94–3.67	<0.0001	2.23	1.52–3.28	<0.0001
Received formal training ≤ 2 years ago	5.92	4.10–8.54	<0.0001	4.93	3.21–7.58	<0.0001
Missing	1.24	0.32–4.88	0.75	1.09	0.18–6.52	0.93
Number of years in practice						
≤10 years	ref			ref		
11–20 years	1.14	0.87–1.50	0.35	0.82	0.57–1.19	0.30
>20 years	2.09	1.57–2.77	<0.0001	1.13	0.70–1.81	0.62
Missing	0.95	0.29–3.14	0.93	0.97	0.21–4.54	0.97
Role						
Family Physician	ref			ref		
Gynecologist	1.35	0.78–2.36	0.28	1.57	0.82–2.99	0.17
Internal Medicine	0.44	0.16–1.17	0.10	0.49	0.15–1.60	0.24
Nurse Practitioner/Advanced Nurse Practitioner	0.53	0.37–0.76	0.0004	0.58	0.37–0.89	0.014
Pediatrician	2.66	1.79–3.95	<0.0001	3.04	1.93–4.78	<0.0001
Physician Assistant	0.24	0.15–0.36	<0.0001	0.27	0.17–0.45	<0.0001
Others	0.15	0.07–0.33	<0.0001	0.23	0.09–0.57	0.002
Facility types or workplace						
University/teaching hospital	ref			ref		
City/County/Public HealthCare facility	1.64	0.87–3.08	0.13	1.34	0.65–2.76	0.43
Employed Physician Practices	1.84	1.09–3.09	0.02	1.33	0.74–2.38	0.33
FQHC/public facility	4.84	2.99–7.84	<0.0001	3.06	1.63–5.75	0.0005
Faith-based hospital/clinic	1.08	0.44–2.64	0.87	1.14	0.41–3.13	0.80
Group practice	2.54	1.88–3.42	<0.0001	1.87	1.28–2.72	0.0012
Others	1.60	0.95–2.69	0.08	1.67	0.88–3.17	0.12
Solo practice	2.30	1.54–3.44	<0.0001	2.33	1.41–3.86	0.001

* We excluded missing data for the outcome variable from the analysis. However, we created a “missing” category for covariates to ensure that these cases were included in the logistic regression analysis.

## Data Availability

The original contributions presented in this study are available upon request. Inquiries can be directed to the corresponding author.

## References

[1] Chesson H.W., Dunne E.F., Hariri S., Markowitz L.E. (2014). The Estimated Lifetime Probability of Acquiring Human Papillomavirus in the United States. Sex. Transm. Dis..

[2] National Cancer Institute HPV and Cancer—NCI. https://www.cancer.gov/about-cancer/causes-prevention/risk/infectious-agents/hpv-and-cancer.

[3] Szymonowicz K.A., Chen J. (2020). Biological and clinical aspects of HPV-related cancers. Cancer Biol. Med..

[4] Damgacioglu H., Sonawane K., Zhu Y., Li R., Balasubramanian B.A., Lairson D.R., Giuliano A.R., Deshmukh A.A. (2022). Oropharyngeal Cancer Incidence and Mortality Trends in All 50 States in the US, 2001–2017. JAMA Otolaryngol. Head Neck Surg..

[5] Cancer Statistics Center. https://cancerstatisticscenter.cancer.org/.

[6] National Cancer Institute USCS Data Visualizations. https://gis.cdc.gov/Cancer/USCS/.

[7] AHR Explore HPV Vaccination in the United States. https://www.americashealthrankings.org/explore/measures/Immunize_HPV.

[8] Pingali C. (2025). Vaccination Coverage Among Adolescents Aged 13–17 Years—National Immunization Survey-Teen, United States, 2024. MMWR Morb. Mortal. Wkly. Rep..

[9] CDC Evidence to Recommendations for HPV Vaccination Harmonization Across Sexes, Ages 22 Through 26 Years. https://www.cdc.gov/acip/evidence-to-recommendations/HPV-harmonization-etr.html.

[10] Meites E., Szilagyi P.G., Chesson H.W., Unger E.R., Romero J.R., Markowitz L.E. (2019). Human Papillomavirus Vaccination for Adults: Updated Recommendations of the Advisory Committee on Immunization Practices. MMWR Morb. Mortal. Wkly. Rep..

[11] US Department of Health and Human Services Vaccination—Healthy People 2030|Odphp.Health.gov. https://odphp.health.gov/healthypeople/objectives-and-data/browse-objectives/vaccination.

[12] CDC Vaccination Coverage Among Adolescents (13–17 Years). https://www.cdc.gov/teenvaxview/interactive/index.html.

[13] Texas DSHS Teens. https://www.dshs.texas.gov/immunizations/data/surveys/nis/teens.

[14] Conrey R., Valencia V., Cioletti A., Williams-Brown M.Y. (2020). Regional variation in human papillomavirus vaccination uptake and completion among adolescents 13–17 in the state of Texas. Vaccine.

[15] Pingali C., Yankey D., Elam-Evans L.D., Markowitz L.E., Valier M.R., Fredua B., Crowe S.J., DeSisto C.L., Stokley S., Singleton J.A. (2023). Vaccination Coverage Among Adolescents Aged 13–17 Years—National Immunization Survey–Teen, United States, 2022. MMWR Morb. Mortal. Wkly. Rep..

[16] Sonawane K., Zhu Y., Damgacioglu H., Garg A., Graboyes E.M., Montealegre J.R., Brownstein N.C., Ford M.E., Roberts J.R., Sterba K.R. (2024). Factors associated with parental human papillomavirus vaccination intentions among adolescents from socioeconomically advantaged versus deprived households: A nationwide, cross-sectional survey. Lancet Reg. Health Am..

[17] Oh N.L., Biddell C.B., Rhodes B.E., Brewer N.T. (2021). Provider communication and HPV vaccine uptake: A meta-analysis and systematic review. Prev. Med..

[18] Hittson H., McAleer L., Saucedo L., Mahler L., Andino G., Zorba A., Walden S., Pickett B.E., Poole B.D., Abel E.L. (2023). Association between Religious Beliefs and HPV Vaccination Attitudes among College Students. Vaccines.

[19] Healy C.M., Montesinos D.P., Middleman A.B. (2014). Parent and provider perspectives on immunization: Are providers overestimating parental concerns?. Vaccine.

[20] Caldwell A.C., Madden C.A., Thompson D.M., Garbe M.C., Roberts J.R., Jacobson R.M., Darden P.M. (2021). The impact of provider recommendation on human papillomavirus vaccine and other adolescent vaccines. Hum. Vaccines Immunother..

[21] Escoffery C., Petagna C., Agnone C., Perez S., Saber L.B., Ryan G., Dhir M., Sekar S., Yeager K.A., Biddell C.B. (2023). A systematic review of interventions to promote HPV vaccination globally. BMC Public Health.

[22] Kaul S., Do T.Q.N., Hsu E., Schmeler K.M., Montealegre J.R., Rodriguez A.M. (2019). School-based human papillomavirus vaccination program for increasing vaccine uptake in an underserved area in Texas. Papillomavirus Res..

[23] Mavundza E.J., Iwu-Jaja C.J., Wiyeh A.B., Gausi B., Abdullahi L.H., Halle-Ekane G., Wiysonge C.S. (2021). A Systematic Review of Interventions to Improve HPV Vaccination Coverage. Vaccines.

[24] Niccolai L.M., Hansen C.E. (2015). Practice- and Community-Based Interventions to Increase Human Papillomavirus Vaccine Coverage: A Systematic Review. JAMA Pediatr..

[25] Rodriguez A.M., Do T.Q.N., Goodman M., Schmeler K.M., Kaul S., Kuo Y.-F. (2019). Human Papillomavirus Vaccine Interventions in the U.S.: A Systematic Review and Meta-analysis. Am. J. Prev. Med..

[26] Smulian E.A., Mitchell K.R., Stokley S. (2016). Interventions to increase HPV vaccination coverage: A systematic review. Hum. Vaccines Immunother..

[27] Walling E.B., Dodd S., Bobenhouse N., Reis E.C., Sterkel R., Garbutt J. (2019). Implementation of Strategies to Improve Human Papillomavirus Vaccine Coverage: A Provider Survey. Am. J. Prev. Med..

[28] CDC Adult Immunization Standards. https://www.cdc.gov/vaccines-adults/hcp/imz-standards/index.html.

[29] Osaghae I., Darkoh C., Chido-Amajuoyi O.G., Chan W., Wermuth P.P., Pande M., Cunningham S.A., Shete S. (2022). Association of provider HPV vaccination training with provider assessment of HPV vaccination status and recommendation of HPV vaccination. Hum. Vaccines Immunother..

[30] Osaghae I., Chido-Amajuoyi O.G., Khalifa B.A.A., Shete S. (2023). Barriers and determinants of consistent offering of HPV vaccination by healthcare facilities. Hum. Vaccines Immunother..

[31] Ramphul R., Zamorano A.S., Upadhyay S., Desai M., Bauer C. (2024). Spatiotemporal analysis of HPV vaccination and associated neighborhood-level disparities in Texas—An ecological study. Front Public Health.

[32] Zhao Y., Zhao J., Xie R., Zhang Y., Xu Y., Mao J., Yan C., Sun Y. (2023). Association between family income to poverty ratio and HPV infection status among U.S. women aged 20 years and older: A study from NHANES 2003–2016. Front. Oncol..

[33] Lin Y.-Y., Damgacioglu H., Suk R., Wu C.-F., Zhu Y., Ortiz A.P., Hara S.K., Sonawane K., Deshmukh A.A. (2022). Trends in the Incidence of Human Papillomavirus-Associated Cancers by County-Level Income and Smoking Prevalence in the United States, 2000–2018. JNCI Cancer Spectr..

[34] Provider Data MasterFile^TM^. https://risk.lexisnexis.com/products/provider-data-masterfile.

[35] Harris P.A., Taylor R., Thielke R., Payne J., Gonzalez N., Conde J.G. (2009). Research electronic data capture (REDCap)—A metadata-driven methodology and workflow process for providing translational research informatics support. J. Biomed. Inform..

[36] Harris P.A., Taylor R., Minor B.L., Elliott V., Fernandez M., O’Neal L., McLeod L., Delacqua G., Delacqua F., Kirby J. (2019). The REDCap consortium: Building an international community of software platform partners. J. Biomed. Inform..

[37] S1901: Census Bureau Table. https://data.census.gov/table/ACSST1Y2022.S1901?g=010XX00US_040XX00US48,48$8600000&t=Income+and+Poverty&y=2022.

[38] SAS Institute Inc SAS. https://www.sas.com/en_us/legal/editorial-guidelines.html.

[39] Chandra M., Osaghae I., Talluri R., Shete S. (2023). Barriers to human papillomavirus vaccine uptake: Role of state religiosity and healthcare professionals’ participation in a state vaccine program. JNCI Cancer Spectr..

[40] Pruitt S.L., Schootman M. (2010). Geographic Disparity, Area Poverty and Human Papillomavirus (HPV) Vaccination. Am. J. Prev. Med..

[41] Do E.K., Rossi B., Miller C.A., Ksinan A.J., Wheeler D.C., Chukmaitov A., Cyrus J.W., Fuemmeler B.F. (2021). Area-Level Variation and Human Papillomavirus Vaccination among Adolescents and Young Adults in the United States: A Systematic Review. Cancer Epidemiol. Biomark. Prev..

[42] Henry K.A., Swiecki-Sikora A.L., Stroup A.M., Warner E.L., Kepka D. (2017). Area-based socioeconomic factors and Human Papillomavirus (HPV) vaccination among teen boys in the United States. BMC Public Health.

[43] CDC About the Vaccines for Children (VFC) Program. https://www.cdc.gov/vaccines-for-children/about/index.html.

[44] Warner E.L., Ding Q., Pappas L.M., Henry K., Kepka D. (2017). White, affluent, educated parents are least likely to choose HPV vaccination for their children: A cross-sectional study of the National Immunization Study—Teen. BMC Pediatr..

[45] CDC (2016). Products—Data Briefs. Number 246. https://www.cdc.gov/nchs/products/databriefs/db246.htm.

[46] Irwin C.E., Adams S.H., Park M.J., Newacheck P.W. (2009). Preventive Care for Adolescents: Few Get Visits and Fewer Get Services. Pediatrics.

[47] Simpson L., Owens P.L., Zodet M.W., Chevarley F.M., Dougherty D., Elixhauser A., McCormick M.C. (2005). Health Care for Children and Youth in the United States: Annual Report on Patterns of Coverage, Utilization, Quality, and Expenditures by Income. Ambul. Pediatr..

[48] Mohammed K.A., Geneus C.J., Osazuwa-Peters N., Boakye E.A., Tobo B.B., Burroughs T.E. (2016). Disparities in Provider Recommendation of Human Papillomavirus Vaccination for U.S. Adolescents. J. Adolesc. Health.

[49] Ellingson M.K., O’Leary S.T., Schwartz J.L., Niccolai L.M. (2025). Influence of Community-Level Factors on Health Care Provider Recommendations for HPV Vaccine. Pediatr. Open Sci..

[50] CDC HPV-Associated Cancer Diagnosis by Age. https://www.cdc.gov/cancer/hpv/diagnosis-by-age.html.

[51] Osaghae I., Darkoh C., Chido-Amajuoyi O.G., Chan W., Padgett Wermuth P., Pande M., Cunningham S.A., Shete S. (2022). HPV Vaccination Training of Healthcare Providers and Perceived Self-Efficacy in HPV Vaccine-Hesitancy Counseling. Vaccines.

[52] Rand C.M., Schaffer S.J., Dhepyasuwan N., Blumkin A., Albertin C., Serwint J.R., Darden P.M., Humiston S.G., Mann K.J., Stratbucker W. (2018). Provider Communication, Prompts, and Feedback to Improve HPV Vaccination Rates in Resident Clinics. Pediatrics.

[53] Dempsey A.F., Pyrznawoski J., Lockhart S., Barnard J., Campagna E.J., Garrett K., Fisher A., Dickinson L.M., O’Leary S.T. (2018). Effect of a Health Care Professional Communication Training Intervention on Adolescent Human Papillomavirus Vaccination: A Cluster Randomized Clinical Trial. JAMA Pediatr..

[54] Yanez B., McGinty H.L., Buitrago D., Ramirez A.G., Penedo F.J. (2016). Cancer outcomes in Hispanics/Latinos in the United States: An integrative review and conceptual model of determinants of health. J. Lat. Psychol..

[55] Villalona S., Stroup A.M., Villalona S., Ferrante J.M. (2022). Racial/ethnic disparities in HPV-related oropharyngeal cancer outcomes among males in the United States: A national cohort study. Ann. Cancer Epidemiol..

[56] Tsegaye A.T., Shing J.Z., Vo J.B., Kreimer A.R., Shiels M.S. (2025). Racial and ethnic differences in HPV-related cancer incidence in the United States, 2001–2020. J. Natl. Cancer Inst..

[57] Gilkey M.B., McRee A.-L. (2016). Provider communication about HPV vaccination: A systematic review. Hum. Vaccin Immunother..

[58] Dombkowski K.J., Leung S.W., Clark S.J. (2008). Physician Perspectives Regarding Annual Influenza Vaccination Among Children With Asthma. Ambul. Pediatr..

[59] Osaghae I., Darkoh C., Chido-Amajuoyi O.G., Chan W., Padgett Wermuth P., Pande M., Cunningham S.A., Shete S. (2023). Healthcare Provider’s Perceived Self-Efficacy in HPV Vaccination Hesitancy Counseling and HPV Vaccination Acceptance. Vaccines.

[60] Palmer J., Carrico C., Costanzo C. (2015). Identifying and Overcoming Perceived Barriers of Providers Towards HPV Vaccination: A Literature Review. J. Vaccines.

[61] Chido-Amajuoyi O.G., Osaghae I., Onyeaka H.K., Shete S. (2024). Barriers to the assessment and recommendation of HPV vaccination among healthcare providers in Texas. Vaccine X.

[62] Gilkey M.B., Grabert B.K., Heisler-MacKinnon J., Bjork A., Boynton M.H., Kim K., Dailey S.A., Liu A., Todd K.G., Schauer S.L. (2022). Coaching and Communication Training for HPV Vaccination: A Cluster Randomized Trial. Pediatrics.

[63] Brewer N.T., Hall M.E., Malo T.L., Gilkey M.B., Quinn B., Lathren C. (2017). Announcements Versus Conversations to Improve HPV Vaccination Coverage: A Randomized Trial. Pediatrics.

[64] Kong W.Y., Oh N.L., Kennedy K.L., Carlson R.B., Liu A., Ozawa S., Brewer N.T., Gilkey M.B. (2024). Identifying healthcare professionals with lower HPV vaccine recommendation quality: A systematic review. J. Adolesc. Health.

[65] Ilyasova A.A., Queen T.L., Gilkey M., Fogel B.N., Odebunmi O.O., Yanguela J., Bamogo A., Patel Y., Laurie E., Ozawa S. (2025). Use of presumptive recommendations and other strategies to encourage HPV vaccine uptake: Results from a national survey of primary care health professionals. PLoS ONE.

[66] Sherry J.S., Collins S.K., McKinnies R.C., Fleege A., Walter M.L. (2018). Human Papilloma Virus and the Nurse’s Role in Education and Prevention. Health Care Manag..

[67] Cook D.A., Wittich C.M., Daniels W.L., West C.P., Harris A.M., Beebe T.J. (2016). Incentive and Reminder Strategies to Improve Response Rate for Internet-Based Physician Surveys: A Randomized Experiment. J. Med. Internet Res..

[68] Asch S., Connor S.E., Hamilton E.G., Fox S.A. (2000). Problems in recruiting community-based physicians for health services research. J. General. Intern. Med..

[69] Tiruneh Y.M., Rachmale R., Elerian N., Lakey D.L. (2024). Assessing Knowledge, Practices, and Barriers to PrEP and nPEP Prescription Among Texas Healthcare Providers. Healthcare.

